# Lingual Concavities in Posterior Mandible: Retrospective Morphometric Analysis in Relation to Gender and Tooth Loss

**DOI:** 10.1155/ijod/5209739

**Published:** 2025-12-22

**Authors:** Khulood Ali Al-Taezi, Wei Chen, Lin Liu, Zhankun Cai, Belal O M Muhaisen, Chunbo Tang

**Affiliations:** ^1^ Department of Oral Implantology, The Affiliated Stomatological Hospital of Nanjing Medical University, Nanjing, 210000, China, njmu.edu.cn; ^2^ State Key Laboratory Cultivation Base of Research, Prevention and Treatment for Oral Diseases, Nanjing, China; ^3^ Jiangsu Province Engineering Research Center of Stomatological Translational Medicine, Nanjing, China

**Keywords:** cone-beam computed tomography, dental implant, gender, lingual concavity, mandible, tooth loss

## Abstract

**Objectives:**

To analyze the posterior mandible bone ridge shape in relation to gender and tooth loss. Focusing on the accompanied changes and variations in lingual concavities.

**Methods:**

406 cone‐beam computed tomography (CBCT) images with dentulous and edentulous 1st and/or 2nd molars (206 females and 200 males, aged ≥20) were examined. Mandibular morphology was classified into U type (undercut), P type (parallel), and C type (convex). The study examined the ridge morphology and dimensions. Lingual concavities angle, area, and the concavity position were measured/reported.

**Results:**

U type bone shape was the most common bone shape 64.1%, then P type was 24.8%, while C type was 11.2 %. U ridge was more frequent in 2nd molar compared to the 1st molar regions (*p* = 0.04), while C type was statistically related to long extraction time (*p* = 0.001). Many bone ridge measurements were higher in males (*p* < 0.05). Other significant differences in measurements were found at 36, 37, 46, and 47 dentate and missing sites (*p* < 0.05). Lingual concavities were larger in males (*p* < 0.001) and relatively larger at 2nd molar dentulous ridges; 37 (*p* = 0.02). Also, they were more statistically frequent at the level of mandibular canal (MC) or above (*p* = 0.0001).

**Conclusion:**

The U type ridge was the most common in the studied population, occurring more often in the second molars region. Bone ridge and lingual concavities dimensions were varied by gender, tooth loss, and specific tooth. Lingual concavities were significantly related to MC level.

**Summary**

 
*What is known:*
• Lingual concavities in the posterior mandible pose risks during dental implant placement, such as lingual plate perforation and nerve damage.
• Studies have classified mandibular ridge morphology into U, P, and C types, but prevalence varies across populations.
• Gender and tooth loss may influence bone ridge dimensions, but findings are inconsistent.
 
*What this study adds:*
• U type ridges are the most common (64.1%) in the studied population, with higher prevalence in second molar regions and males showing larger dimensions and lingual concavities.
• Tooth loss significantly impacts bone ridge morphology over time, with C type ridges associated with long‐term extraction.

## 1. Introduction

The accurate assessment of mandibular anatomy is crucial in various dental and surgical procedures, particularly those involving the placement of dental implants in the posterior mandible. One of the significant anatomical features in this region is the mandibular lingual concavity, a depression on the lingual side of the mandible that poses a potential risk during surgical interventions [1, 2]. The complex anatomy of the mandibular lingual concavities, particularly in the posterior region, presents unique challenges that must be carefully navigated to ensure successful implant placement and long‐term outcomes. These variations are critical, as they directly influence the choice of implant size and angulation. A thorough understanding of these variations can help clinicians avoid complications such as lingual plate perforation, which could lead to severe complications, including hemorrhage or nerve damage [3].

Mandible ridge morphology has been thoroughly studied [4, 5] for the purpose of adequate diagnosis and presurgical planning. Mandibular cross‐sections were identified in which the anatomical information needed for ideal alignment of dental implants can be determined. According to the cone‐beam computed tomography (CBCT) cross‐sectional morphology, alveolar ridge classified into different classes [6]. The classification most frequently used in literature is the one introduced by Chan et al. [5]. It categorizes the bone ridge into three types: convergent (C) type, parallel (P) type, and undercut (U) type. The prevalence of bone shape varies among different studies. Some studies have found that the U‐type ridge can be as prevalent as 93% [7], while others have reported a higher occurrence of the C type ridge, with a prevalence of 51.7% [8]. However, the complex morphology of the mandibular lingual concavity requires careful consideration and detailed analysis to ensure patient safety and optimal outcomes. Traditional two‐dimensional (2D) imaging techniques often fall short in accurately depicting the three‐dimensional (3D) nature of this feature, potentially leading to underestimation of its dimensions. With the advent of advanced 3D imaging technologies, such as CBCT, clinicians now have access to more precise and comprehensive views of mandibular anatomy [9, 10].

Despite these technological advancements, detailed studies that quantitatively and qualitatively analyze bone ridge and lingual concavity are needed. Understanding dimensions and variations across different populations can significantly enhance the planning and execution of dental implant procedures. Moreover, identifying factors such as gender, age, and extraction history that influence the morphology of the lingual concavity can provide valuable insights for specific treatment approaches.

This study aims to enrich the literature by conducting an in‐depth analysis of the mandibular ridge focusing on lingual concavities using a 3D imaging technique. By examining the type and dimensions, along with their implications for implant placement, this research seeks to improve the safety and efficacy of dental and surgical procedures in the posterior mandible.

## 2. Materials and Methods

In this cross‐sectional study, CBCT scans of patients were obtained between 2020 and 2024 by a search of the electronic health records at the Jiangsu stomatological affiliated hospital of Nanjing Medical University. The Affiliated Stomatological Hospital of Nanjing Medical University Ethical Committee Department approved retrieval and assessment of the CBCT scans for this study (Approval # PJ2020‐130‐001). The study conducted with the guidelines laid out in the Declaration of Helsinki. Any patient’s data that can potentially breach data protection rules and regulations was exempted.

### 2.1. Sample

#### 2.1.1. Sample Size

The population was an accessible population (Chinese people). Sample population was initially > 10,000. Sample size was calculated based on Cochran’s formula [11]:

n=z2p1−pd2,

*z* = *z* score or standards of normal distribution at 95% confidence level.

z=1.96,

*p* = prevalence of U type in pervious literature.

U type prevalence selected as it was recorded in the literature [4].

p=46.3%,

d=Margin of error 5%,

n=1.9620.4630.5370.052,

n≥382.05.

Minimum size of sample ≥383 subject.

#### 2.1.2. Participants

The enrolled patients were all referred for CBCT scans by dentists in the implantology department to the radiologists for implant treatment planning purposes.

All CBCTs evaluated with inclusion criteria are as follows:

1. Aged ≥20 years old.
2. High‐quality images without defects due to motion, metal artifacts, or any other reasons.
3. Partially edentulous posterior mandible (for each missing 1st or 2nd molar, a corresponding molar is present on the other side).

Exclusion criteria:

1. Systemic endocrine diseases that influence bone metabolism (e.g., hyperparathyroidism, Paget’s disease, and osteoporosis).
2. Any local condition that may affect bone quantity and quality at the posterior mandible (e.g., periodontal diseases, cysts, trauma, or surgery) was excluded.

#### 2.1.3. Image Acquisition

Imaging parameters set at 110 kVp, 5 mA, scan time 20 s and resolution 0.3 mm in the VGi EVO scanning machine (Newtom, Verona, Italy). Patients’ head positions for images were unified according to the lines created by the manufacturer. CBCT images were loaded into Anatomage Invivo 7.0 virtual implant planning software (Anatomage, San Jose, CA, USA). Each CBCT volume was reoriented so that the mandibular occlusal plane was parallel to the horizontal reference plane and the midsagittal plane was centered. Cross‐sectional slices were taken perpendicular to the alveolar ridge at the middle of the molar site to ensure consistency across all slices. All measurements were performed under identical display settings (same monitor resolution, contrast, and brightness). Images were viewed in a dimly lit area on a 19‐inch flat panel screen with a 1920^∗^1080‐pixel resolution (HP Development Company, Palo Alto, Calif).

All morphologic assessments and measurements were conducted by two examiners. The intraexaminer agreement was determined by comparing two repeated measurements at randomly chosen sites taken 1 week apart. The intraexaminer agreement was assessed using the intraclass correlation coefficient (ICC), yielding an ICC value of 0.82, indicating good reliability.

### 2.2. Categorization of Posterior Mandibular Bone Cross‐Sections

According to Chan et al. [5], the morphology of bone ridge cross‐sections was divided into three categories (Figure 1):

Figure 1Different bone ridge morphology. Different cross‐sections in anterior–posterior (AP) view or (¾) left (d) or right (e, f) lateral views reveal different bone morphology, U type (a, d), P type (b, e), and C type (c, f).

**Figure:**
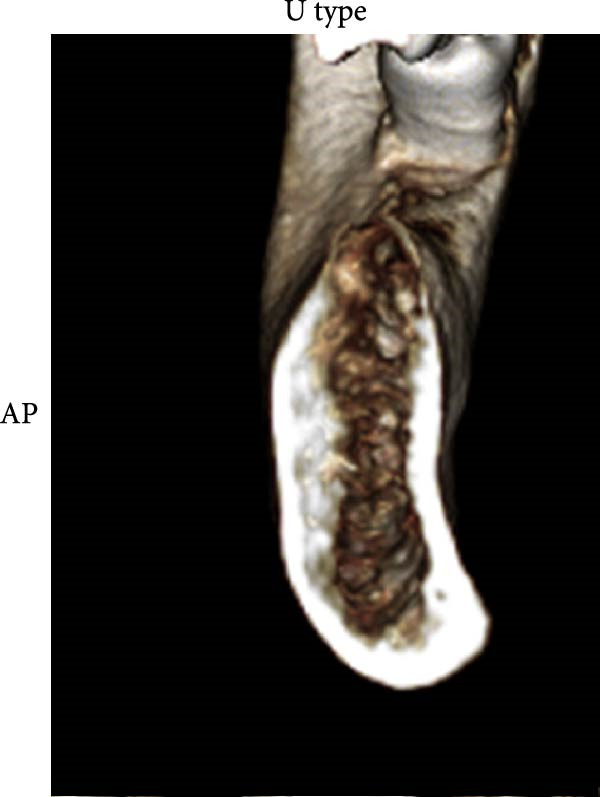
(a)

**Figure:**
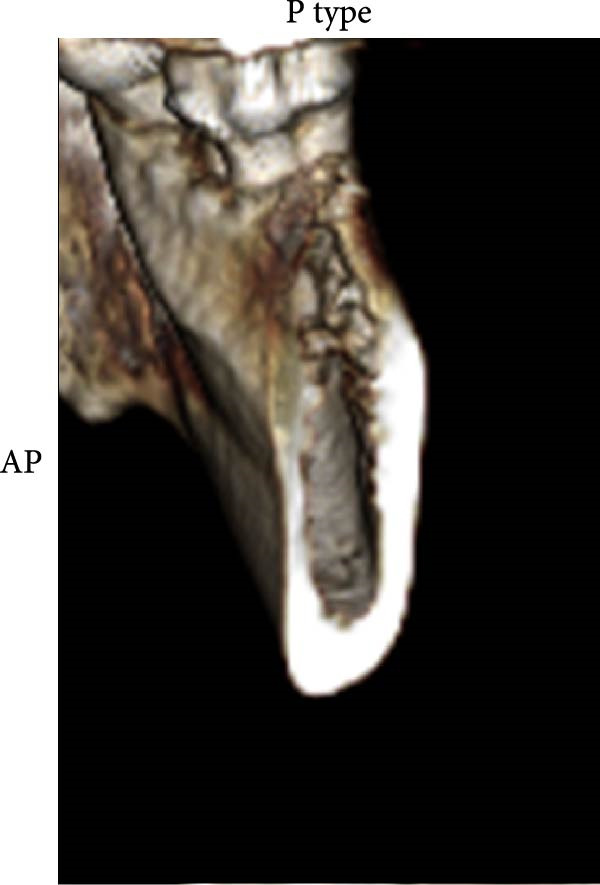
(b)

**Figure:**
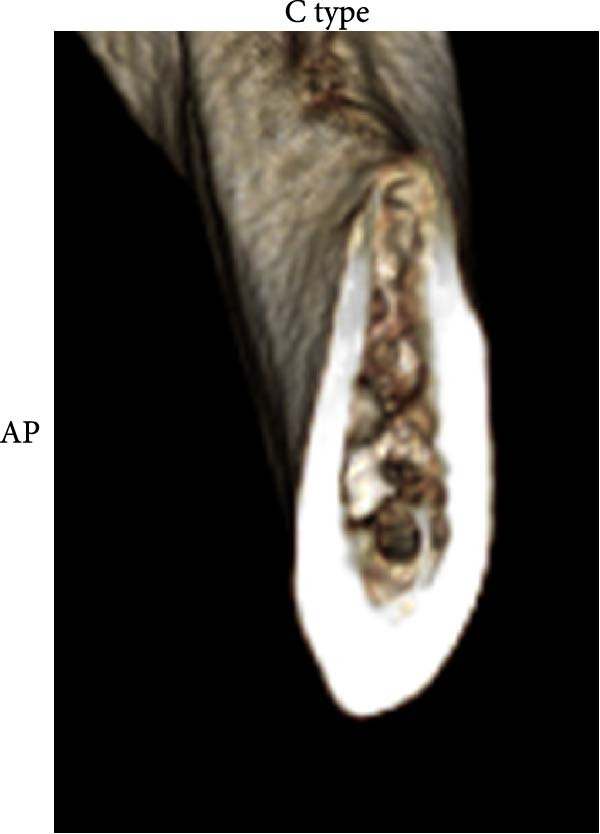
(c)

**Figure:**
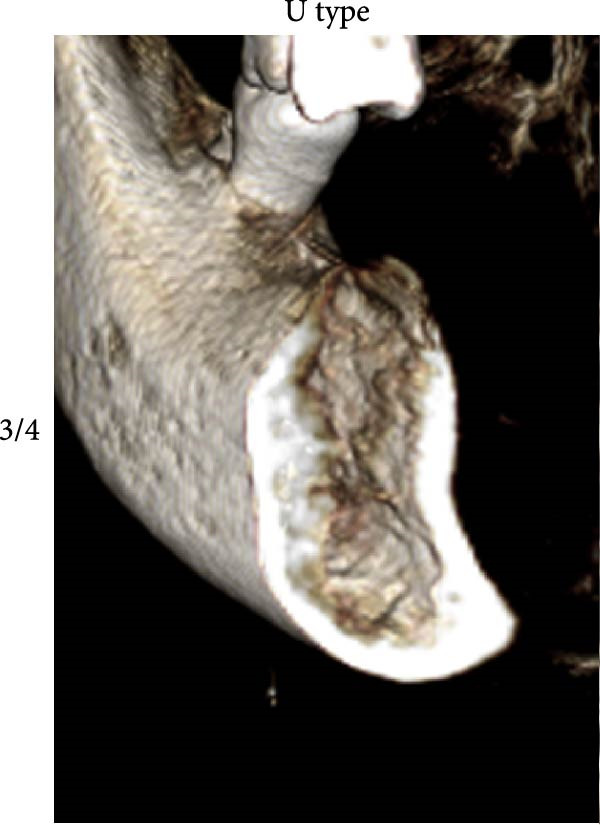
(d)

**Figure:**
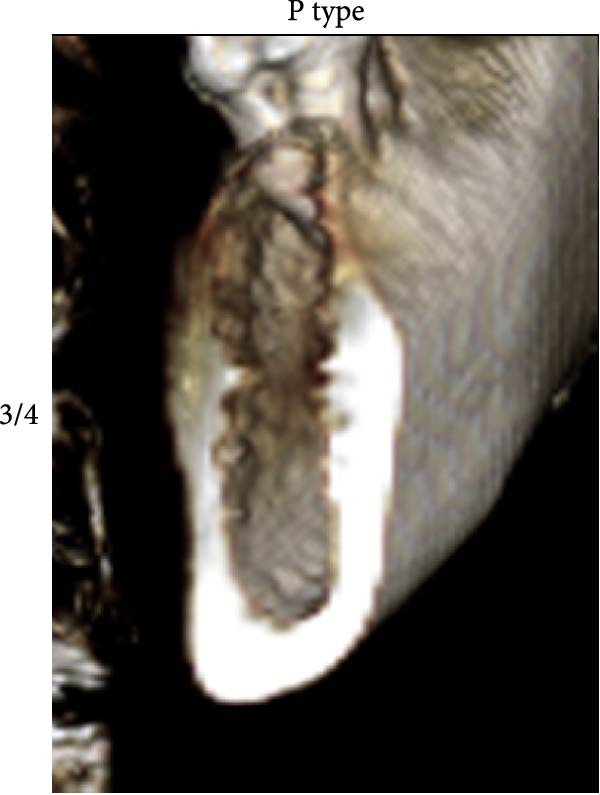
(e)

**Figure:**
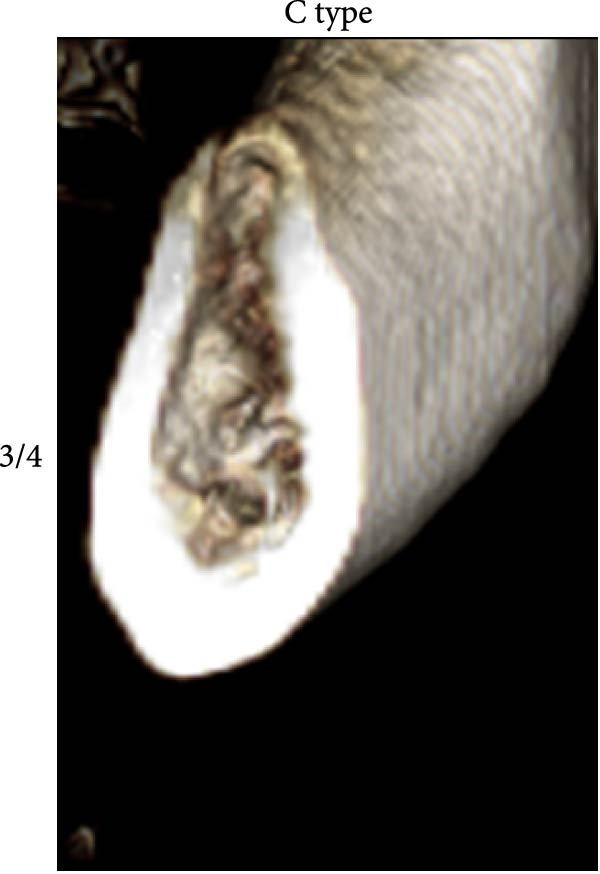
(f)

1. The undercut type (Class U): When the bone ridge has a concavity at the lingual side, known as the U type (Figure 1a, d).
2. The parallel type (Class P): When buccal and lingual walls nearly align parallel regardless of ridge inclination to buccal or lingual directions (Figure 1b, e).
3. The convex type (Class C): The alveolar bone shape tends to be wider at the base more than the crest (Figure 1c, f).

### 2.3. Statistical Analysis

The three types of bone shapes were crossly tabulated. Spearman correlation coefficients between bone classes and gender, sites and, right and left sides were estimated. Generally, *t*‐tests were performed for dimensions statistics; two‐sample *t*‐test was used to compare statistical differences between male and female dimensions. The chi‐square test was used to assess the relationship between bone classes and extraction time groups. Different tooth sites (36, 37, 46, and 47) in missing and dentate regions were compared using one‐way analysis of variance (ANOVA) with a post hoc paired *t*‐test. The degree of concordance estimated by a weighted kappa statistic and 95% confidence interval (CI) and the statistical significance set at *p* value < 0.05. SPSS 29.0 software (SPSS, Chicago, IL) was used for the statistical analysis.

### 2.4. Outcomes

#### 2.4.1. Qualitative Criteria

Distribution of different bone ridge shapes in the posterior mandible, patient’s attributes; gender, tooth site, and tooth loss history.

#### 2.4.2. Quantitative Criteria

a. As shown in Figure 2a, the horizontal dimensions, “W1,” “W3,” and “W5” were representing the buccolingual width of the ridge measured 1 mm, 3 mm, and 5 mm below the bone crest, respectively. Horizontal lines named “Wmini” and “Wmax” were drawn at the minimum and maximum buccolingual widths of the ridge. “WMC” was represented the buccolingual width at the level of mandibular canal (MC). Longitudinal dimensions were as the following: “HT tot,” the total ridge height, was taken from the highest superior point on the bone ridge to the most inferior point on the mandibular border of the ridge vertically for the edentulous ridge. On the other side, the dentulous ridge was measured from the highest point of the alveolar crestal bone around the tooth to the most inferior point of the ridge vertically. Ridge height regarding MC; “HTMC” was created following the same pattern of “HT tot” from the ridge crest and ended at the “WMC” line. Ridge length “LTMC” was drawn mimicking the implant axis (Figure 2b).
b. For‐U type ridge, the concavity angle (CAn) and concavity area (CAr) were recorded. The CAn was obtained by drawing an angle with its vertex at the deepest point of the lingual concavity (point B), one ray passing through the most convex point of the lingual bone plate coronally (point A), and the other ray being a horizontal line parallel to the inferior mandibular border (Figure 2c). To determine CAr, a straight line was drawn from point A to the most convex point of the lingual bone plate apically (point D). The examination software then calculated the involved area using its built‐in function (Figure 2d).
c. Concavity position: The position of the concavity and its relationship to MC were evaluated by identifying the deepest or steepest concavity. It was classified according to its relation to MC into five groups as shown in (Figure 3).

Figure 2Horizontal and longitudinal bone ridge dimensions. (a) W1, W3, and W5 (green lines) represent buccolingual width at 1 mm, 3 mm, and 5 mm depth, respectively. W max and W mini (red lines) represent the maximum and minimum buccolingual widths for the bone ridge. WMC (blue line) represents buccolingual width at the MC level. (b) HTMC is the vertical bone ridge height from the crest to WMC, while HT tot is the total height from the crest to the inferior mandibular border vertically. LTMC is the bone ridge length from the crest to WMC in the implant axis direction. (c) Concavity angle CAn, an angle with its vertex at the deepest point of the lingual concavity (point B), one ray passing through the most convex point of the lingual bone plate coronally (point A), and the other ray is a horizontal line passing through (point B) and parallel to the mandibular line. (d) A straight line was drawn from point A to the most convex point of the lingual bone plate apically (point D); the area involved in the lingual concavity represents the concavity area CAr.

**Figure:**
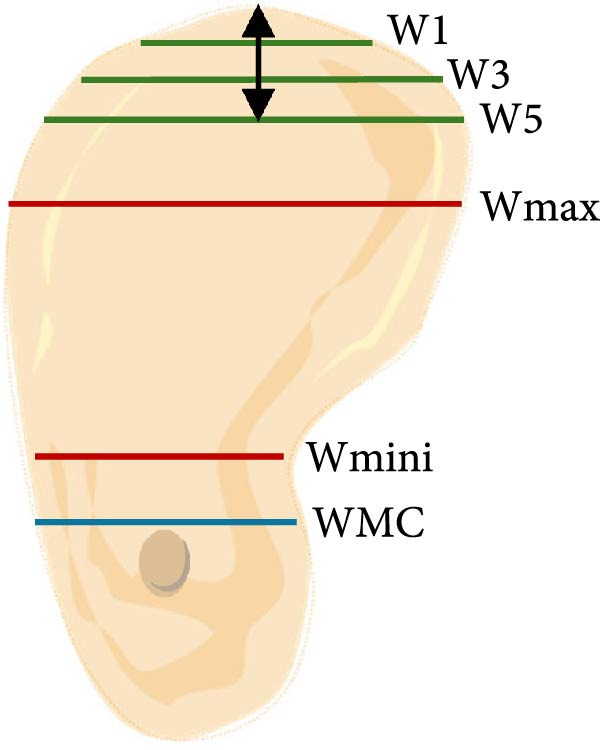
(a)

**Figure:**
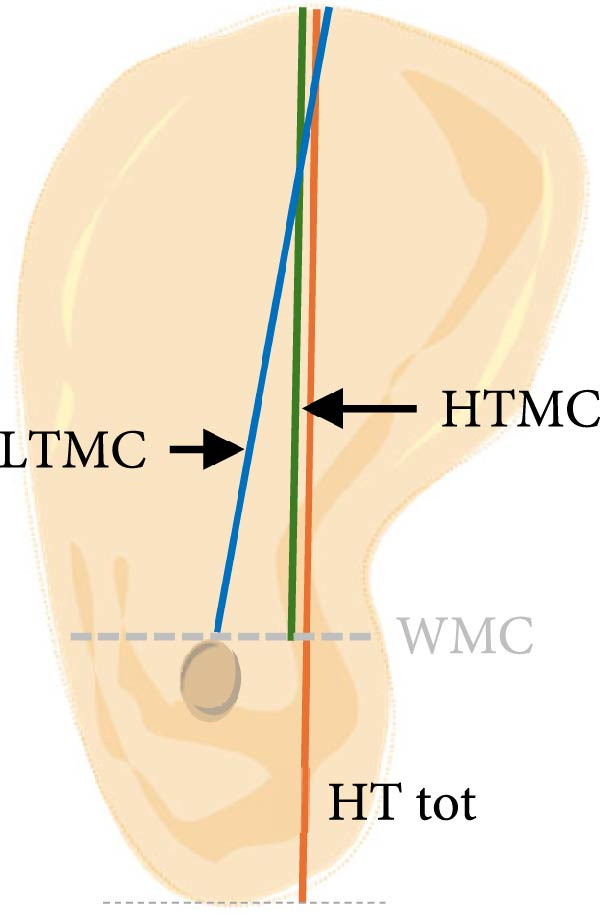
(b)

**Figure:**
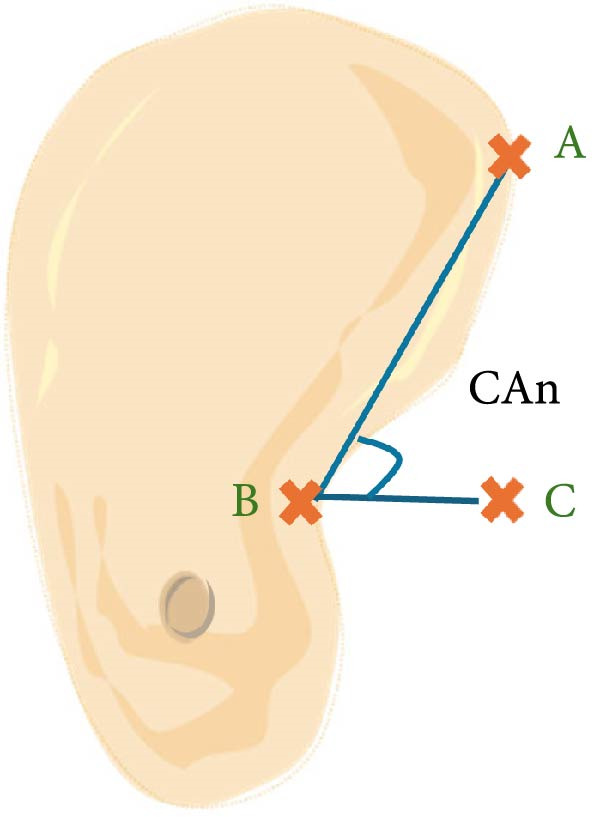
(c)

**Figure:**
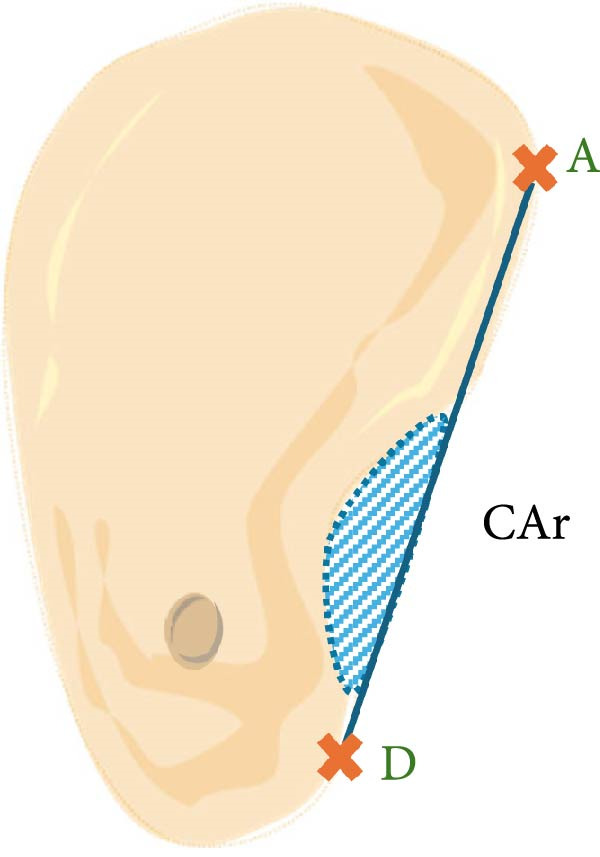
(d)

Figure 3Different lingual concavity positions in relation to MC. (a) The most concave area is above the MC level, (b) under MC and concave, (c) under MC and straight, (d) in line w/MC and concave, and (e) in line w/MC and straight.

**Figure:**
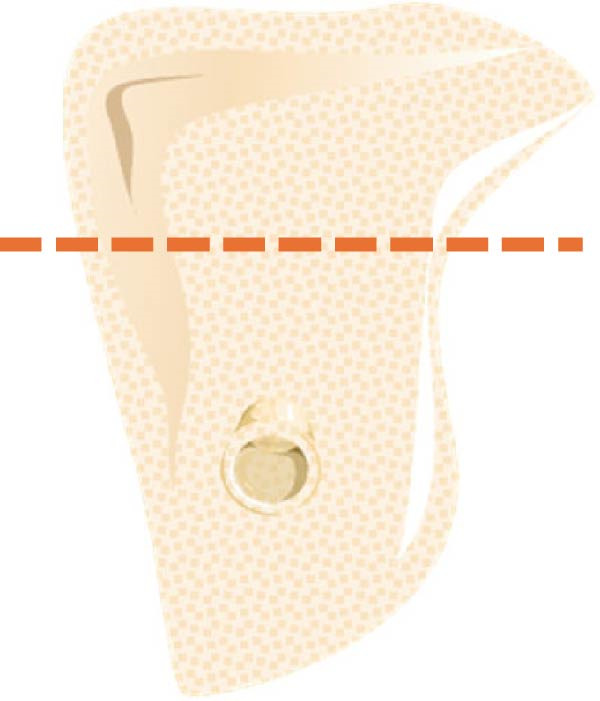
(a)

**Figure:**
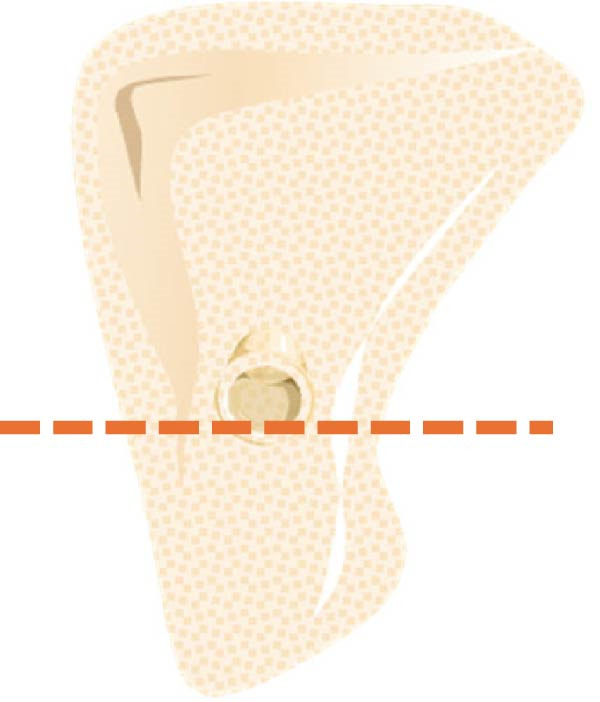
(b)

**Figure:**
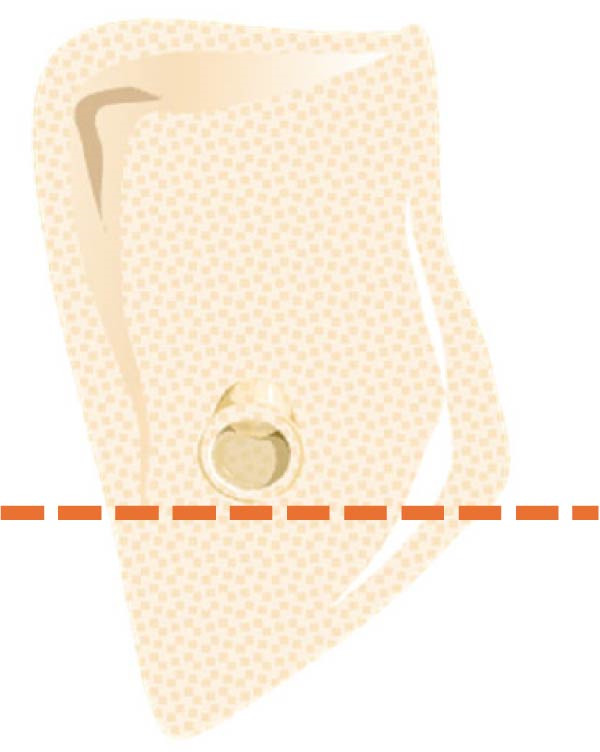
(c)

**Figure:**
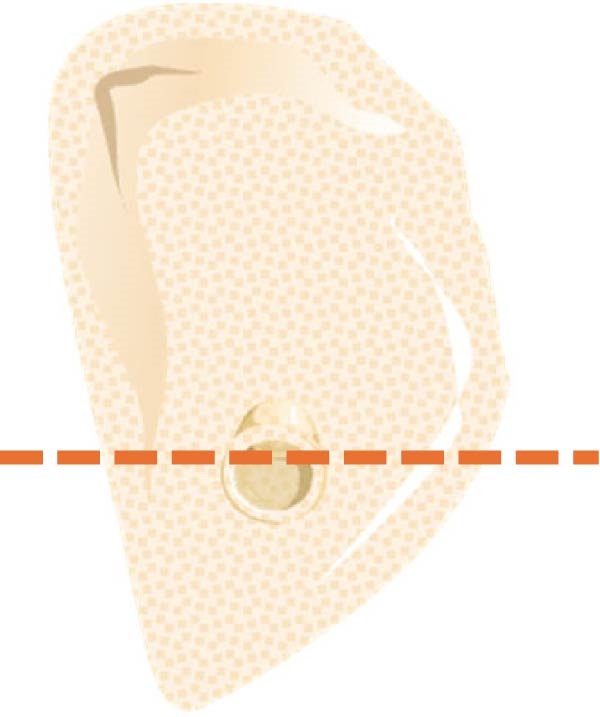
(d)

**Figure:**
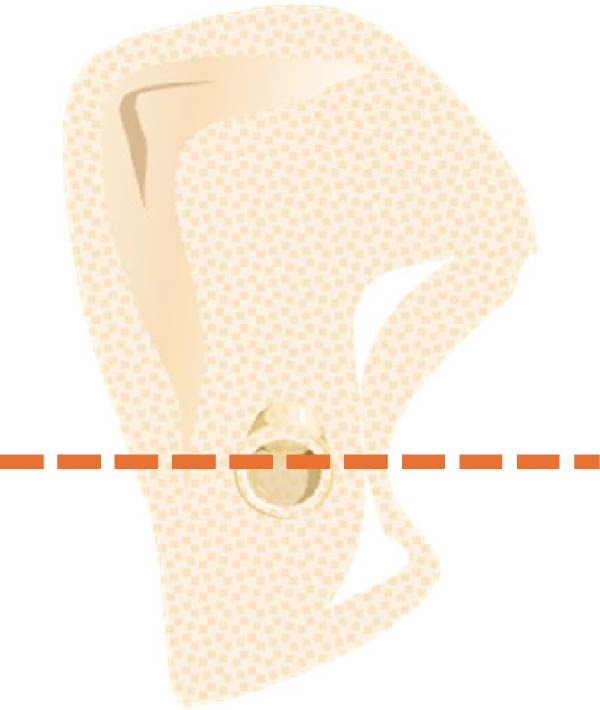
(e)

## 3. Results

### 3.1. Qualitative Analysis

In total, 406 CBCT images were collected with dentate and missing mandibular first or second molars. The average age of patients was 47.68 ± 14.97 years with a range from 20 to 79 years. Amongst 206 females (F, 51.4%) and 200 males (M, 48.6%). The U type was the most common bone type; presented in 64.1% (*n* = 263), then P type in 24.8% (*n* = 98), and the type C was only in 11.1 % (*n* = 45). Spearman correlation coefficient between bone shape variations and gender was not statistically significant (Table 1). 1st molar sites had a higher prevalence of P type (19.1% vs. 2.9%) and C type (10.2% vs. 1.9%) ridges than 2nd molar sites. Nonetheless, U type ridges were the most type prevalent in 2nd molar sites (37.2%) and amounted to 28.4% in 1st molar sites (*p* value 0.04). Tooth side correlation to bone morphology were not statistically significant as depicted in Table 1. Extraction time was divided into five groups in months (Table 1). These groups were significantly related to bone morphology, with C type being associated to long‐term tooth extraction (*p*  = 0.001).

**Table 1:** *p* Values for bone morphology and different patient’s criteria.

Pateint criteria	*N*	*p* Value
Side		
Right	207	0.07
Left	199	
Total	406	

Tooth site		
1st molar	257	0.04**^∗^**
2nd molar	149	
Total	406	

Gender		
Male	200	0.51
Female	206	
Total	406	

Extraction time		
3–6 months	74	0.001**^∗^**
7–12 months	54	
13–24 months	47	
25–60 months	45	
> 60 months	61	
Total	281	

^∗^Significant difference between different bone shapes occurrence and patient criteria (*p* value < 0.05).

### 3.2. Quantitative Analysis

The descriptive statistics for these measurements were as below:

#### 3.2.1. Horizontal Measurements

**Figure 4:**
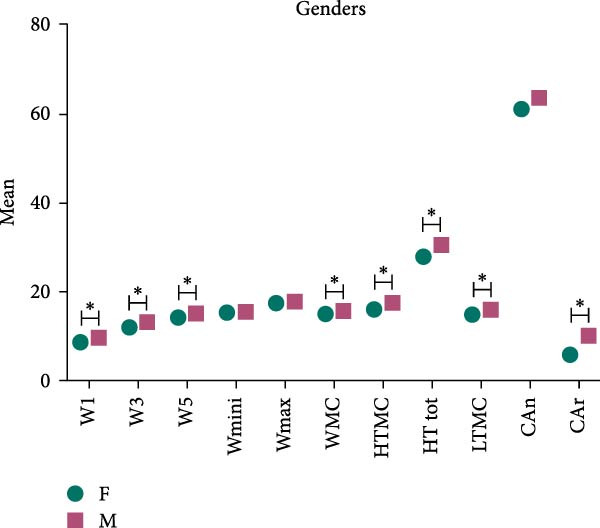
Alveolar ridge dimensions and gender differences were significant at W1, W3, W5, WMC, HTMC, HT tot, and CAr (*p* value < 0.05).

i. W1: W1 mean was 7.44 ± 3.62 mm, with a range (3.02–18.99) mm. (8.71 ± 3.5 mm in females vs. 9.71 ± 3.6 mm in males, *p* = 0.01, Figure 4). Significant differences were also observed between dentate and missing areas in all molars sites (*p* values were illustrated in Tables 2 and 3).
ii. W3: W3 mean 11.24 ± 3.53 mm, with a range (6.33–21.30) mm. (12.07 ± 3.5 mm in females vs. 13.27 ± 3.4 mm in males, *p* = 0.001, Figure 4). The mean difference between dentate and missing areas was statistically significant at right 1st and 2nd molars, and left 2nd molar (*p* = 0.0001, *p* = 0.01, and *p* = 0.01), respectively.
iii. W5: W5 mean was 13.86 ± 3.19 mm, with a range (8.41–22.53) mm. (14.17 ± 3.1 mm in females vs. 15.30 ± 3.0 mm in males, *p* = 0.0003, Figure 4). For dentate and missing areas means differences were statistically significant at right 1st, and 2nd molars and left 1st molars; *p* values were 0.002, 0.02, and 0.03 in row.
iv. W mini: W mini was 15.34 ± 2.59 mm, with a range (7.78–23.33) mm. (15.33 ± 2.3 mm in females vs. 15.56 ± 3.0 mm in males, *p* = 0.61, Figure 4). No statistically significant differences were found regarding dentate and missing areas (Tables 2 and 3).
v. Wmax: Wmax mean was 17.51 ± 2.50 mm, with a range (11.11–23.88) mm. (17.52 ± 2.4 mm in females vs. 17.91 ± 2.7 mm in males, *p* = 0.34, Figure 4). The difference between dentate and missing areas was statistically significant at right 1st molar (*p* = 0.01).
vi. WMC: The mean was 15.33 ± 2.66 mm, with a range (8.00–23.33) mm. (15.03 ± 2.2 mm in females vs. 15.80 ± 2.7 mm in males, *p* = 0.002, Figure 4). There was no significant statistical difference between dentate and missing areas (Tables 2 and 3).

**Table 2:** Comparing means in dentate and missing sites for 36, 37, 46, and 47.

36 *n* = 122	Mean of dentate (mm)	Std. deviation	Mean of missing (mm)	Std. deviation	Difference	Std. error mean	*p* Value	37 *n* = 77	Mean of dentate (mm)	Std. deviation	Mean of missing (mm)	Std. deviation	Difference	Std. error mean	*p* Value
W1	10.22	1.53	7.08	3.65	3.13	0.79	0.0004 ^∗^	—	11.50	2.74	8.42	3.18	3.08	1.14	0.02 ^∗^
W3	11.50	2.74	10.07	3.49	1.43	0.77	0.08	—	15.44	2.57	12.70	3.14	2.74	0.96	0.01 ^∗^
W5	13.94	2.32	12.45	3.04	1.49	0.66	0.03**^∗^**	—	17.04	2.37	16.15	2.35	0.89	0.65	0.19
Wmini	14.54	2.36	13.83	2.71	0.7	0.99	0.49	—	16.25	2.26	15.22	2.59	1.03	0.9	0.27
Wmax	16.77	2.22	16.96	2.23	−0.19	0.69	0.79	—	18.37	2.16	18.60	1.79	−0.22	0.71	0.75
WMC	14.35	2.38	14.62	2.38	−0.27	0.54	0.62	—	16.74	2.29	15.23	2.56	1.51	0.81	0.08
HTMC	30.20	18.88	28.93	19.98	1.27	0.84	0.14	—	28.60	13.90	28.46	10.68	0.15	1.58	0.92
HT tot	17.47	2.37	16.71	2.73	0.76	0.71	0.29	—	15.81	3.35	16.41	2.56	−0.59	1.05	0.58
LTMC	16.32	2.22	15.76	2.75	0.56	0.69	0.42	—	14.20	3.21	15.06	2.90	−0.85	1.08	0.44
CAn (x°)	66.68	3.16	60.15	3.62	6.54	8.07	0.43	—	59.45	4.24	60.18	3.63	−0.72	5.72	0.9
CAr (mm^2^)	9.59	8.75	9.73	7.18	−0.13	2.11	0.95	—	13.54	3.43	7.533	8.59	6.005	2.294	0.02**^∗^**

**46 *n* = 135**	**Mean of dentate (mm)**	**Std. deviation**	**Mean of missing (mm)**	**Std. deviation**	**Difference**	**Std. error mean**	***p* Value**	**47 *n* = 72**	**Mean of dentate (mm)**	**Std. deviation**	**Mean of missing (mm)**	**Std. deviation**	**Difference**	**Std. error mean**	***p* Value**

W1	10.57	3.00	6.45	2.25	4.12	0.66	<0.001 ^∗^	—	12.69	2.96	9.43	5.16	3.25	1.49	0.04 ^∗^
W3	13.63	3.20	10.32	2.72	3.31	0.76	0.0001 ^∗^	—	16.53	2.22	13.60	4.04	2.92	1.01	0.01 ^∗^
W5	15.27	2.94	12.87	2.66	2.4	0.73	0.002 ^∗^	—	17.96	1.65	16.20	2.81	1.76	0.71	0.02 ^∗^
Wmini	15.09	2.54	14.46	1.97	0.63	1.41	0.66	—	15.74	2.83	16.82	2.92	−1.08	1.15	0.36
Wmax	19.10	2.56	16.32	2.46	2.77	0.87	0.01 ^∗^	—	18.77	1.55	18.96	2.26	−0.19	0.83	0.81
WMC	15.78	2.05	14.97	2.43	0.81	0.58	0.17	—	15.87	2.52	17.50	2.77	−1.62	0.92	0.09
HTMC	29.57	14.25	29.49	17.65	0.07	0.72	0.91	—	30.45	17.01	26.99	24.29	3.46	1.55	0.01 ^∗^
HT tot	17.59	3.04	17.19	2.91	0.39	0.52	0.45	—	17.51	3.36	14.63	3.13	2.88	1.10	0.03 ^∗^
LTMC	16.33	3.04	15.87	2.95	0.46	0.62	0.46	—	15.01	3.20	12.83	3.54	2.18	1.27	0.1
CAn (x°)	67.98	3.71	68.42	3.30	−0.44	6.09	0.94	—	61.99	4.10	54.82	4.34	7.17	8.71	0.42
CAr (mm^2^)	6.9	4.69	8.03	4.42	−1.12	3.03	0.72	—	11.98	10.26	4.73	3.83	7.24	3.44	0.05

*Note*: W1, W3, and W5 are buccolingual width at 1 mm, 3 mm, and 5 mm depth, respectively. Wmax and Wmini are the maximum and minimum buccolingual widths for the bone ridge. WMC is buccolingual width at MC level. HTMC is the vertical bone ridge height to WMC. HT tot is the total ridge height vertically. LTMC is the bone ridge length to WMC in implant axis direction.

Abbreviations: CAn, concavity angle; CAr, concavity area.

^∗^Significant difference in means with greater values in dentate than missing sites, statistical significance set at (*p* value < 0.05).

**Table 3:** Comparing means in dentate and missing sites for the same individual.

Parameter	Mean	Std. deviation	*N*	Mean difference	Std. deviation	Std. error mean	95% Confidence interval of the difference		*p* Value
							Lower	Upper	
W1_Dentate W1_Missing	10.41	3.22	406	2.43	3.82	0.38	1.68	3.17	<0.001 ^∗^
	7.98	3.64							
W3_Dentate W3_Missing	13.66	3.27	406	2.00	3.52	0.35	1.32	2.69	<0.001 ^∗^
	11.65	3.57							
W5_Dentate W5_Missing	15.30	3.00	406	1.17	2.94	0.29	0.60	1.74	<0.001 ^∗^
	14.13	3.19							
Wmini_Dentate Wmini_Missing	15.22	2.29	263	−0.07	1.54	0.21	−0.49	0.34	0.725
	15.29	2.28							
Wmax_Dentate Wmax_Missing	17.53	2.45	263	−0.32	2.13	0.29	−0.90	0.25	0.265
	17.86	2.09							
WMC_Dentate WMC_Missing	15.44	2.51	406	0.08	1.74	0.17	−0.26	0.42	0.634
	15.36	2.58							
HTMC_Dentate HTMC_Missing	17.13	3.02	406	0.64	2.48	0.24	0.15	1.12	0.011 ^∗^
	16.49	2.90							
LTMC_Dentate LTMC_Missing	15.65	3.05	406	0.44	2.42	0.24	−0.03	0.92	0.066
	15.20	3.04							
HTtot_Dentate HTtot_Missing	29.72	3.65	406	0.94	1.93	0.19	0.56	1.32	<0.001 ^∗^
	28.78	3.77							
CAn_Dentate CAn_Missing	64.73	17.07	263	4.29	17.60	2.40	−0.52	9.09	0.079
	60.44	16.62							
CAr_Dentate CAr_Missing	8.07	7.51	263	−0.68	6.38	0.88	−2.44	1.08	0.441
	8.76	6.99							

*Note*: W1, W3, and W5 are buccolingual width at 1 mm, 3 mm, and 5 mm depth, respectively. Wmax and Wmini are the maximum and minimum buccolingual widths for the bone ridge. WMC is buccolingual width at MC level. HTMC is the vertical bone ridge height to WMC. HT tot is the total ridge height vertically. LTMC is the bone ridge length to WMC in implant axis direction.

Abbreviations: CAn, concavity angle; CAr, concavity area.

^∗^Significant difference in means with different values in dentate and missing sites, statistical significance set at (*p* value < 0.05).

#### 3.2.2. Longitudinal Measurements

i. HTMC: HTMC mean was 16.39 ± 2.92 mm, with a range (10.43–23.35) mm. Means in females (16.05 ± 2.5 mm) and males (17.61 ± 3.1 mm) were statistically different (*p* < 0.001, Figure 4) while no statistical difference was found between dentate and missing areas (Tables 2 and 3).
ii. HT tot: HT tot was 28.68 ± 3.69 mm, with a range (18.46–35.18) mm. HT tot mean value was 27.92 ± 3.0 mm in females and 30.65 ± 3.8 mm in males (*p* < 0.001, Figure 3). The difference between dentate and missing areas was not statistically significant (Tables 2 and 3).
iii. LTMC: LTMC mean was 15.11 ± 3.14 mm, with a range (7.46−22.43) mm. it was 14.68 ± 2.7 mm in females and 16.02 ± 3.2 mm in males (*p* = 0.0001, Figure 4). The difference between dentate and missing areas was not statistically significant (Tables 2 and 3).
iv. CAn: The mean angle was 61.2 ± 8.9°, with a range (35.3°−78.6°). CAn mean value was 61.17 ± 19.9° in females and 63.70 ± 15.6° in males (Figure 4). The difference between dentate and missing areas was also not significant (Tables 2 and 3).
v. CAr: CAr mean was 8.36 ± 7.5, ranging from 0.47 to 28.56 mm [2]. CAr was 6.25 ± 4.4 and 10.18 ± 8.6 in females and males respectively (Figure 4). There was significant statistical difference between dentate and missing in right 2nd molar sites (Table 2).
vi. Concavity position: The prevalence of different concavity positions among U‐shaped cases was statistically significant (). The prevalence was as follows: above MC 3.6%, under MC and concave 2.6%, under MC and straight 20.1%, in line w/MC and concave 22.7%, and in line w/MC and straight 15% (*n* = 263).

## 4. Discussion

The most severe complications in posterior mandible mentioned in the literature are those happen intraoperatively [12]. Misjudgment of the lingual concavity can result in severe intraoperative complications, including lingual plate perforation, hemorrhage, and damage to vital anatomical structures [13]. According to the findings of this study, the decision of implant placement is based on multiple properties, such as patient criteria and site conditions which impose appropriate treatment approaches to reduce associated surgical risks. The cross‐sectional bone morphology has been previously classified according to shape of the alveolar ridge into C, P, and U ridge types [5]. Accordingly, we did sort out and analyzed different bone shapes prevalence in our sample. Present study findings contrasted with those reported by Tan et al. [8] in which the common bone shape was C type 51.7%, while in our study U type was the common type made up 64.1% of the total sample. This result came along with results yielded by other researchers [13, 14], as well. The anatomical structure of posterior mandible relatively explained these findings.

As for gender differences, previous studies have shown results like the present study, indicating no significant relationship between gender and the prevalence of different bone shapes [15]. However, other studies have reported different findings [16] showing prevalent U ridge at 1st molar site in males. Regarding the tooth site, the predominance of the U type in 2nd molar sites (37.2%) compared to 1st molar sites (28.4%) was consistent with other researches [14, 16] indicating that the anatomical structure of the mandible near the 1st molar site typically presents a shallow and wide concavities compared to 2nd molar which are steep and deep. For alveolar bone ridge width, our study showed significant differences at different horizontal measurements among different genders. Along with different studies [5, 10], males showed wider alveolar bone ridge than females; however, Alqutaibi et al. [16] showed smaller ridge width in males than females (Table S1). While bone ridge was consistently longer in males than females in the present study and previous studies (Table S2). According to Al‐jabrah et al. [17] study, women are at more risk to have ridge resorption compared to men. Furthermore, Coquerelle et al. [18] mentioned, from puberty to adulthood, males experience mandibular changes that proportionate to their size increase, whereas the changes of the female mandible continue even after growth in size has stopped. These findings would provide an interpretation for dimension diversity between genders in posterior mandible.

In our study extraction time played a significant role in which the bone ridge progressively transformed into a C shape over time. This could be explained due to bone resorption process [19]. Additionally, variations in results between dentate and missing sections (W1, W3, and W5) explained the preceding outcome. Comparing dentulous and edentulous sites for the same individual, the data suggests tooth loss significantly affects some bone morphometric measurements (e.g., W1, W3, W5, HTMC, and HT tot). However, for other parameters like Wmini, Wmax, and WMC, CAn, and CAr, the differences are not statistically significant, meaning tooth loss may not have a strong effect on parameters. As well as other studies, dentulous sites exhibit significant higher bone ridge and greater width [10, 20]. Mandibular bone ridge dimensions often vary between dentulous and edentulous sites due to differences in functional load and remodeling processes. When teeth are present, the alveolar bone is maintained by the mechanical forces exerted during mastication [21]. In contrast, the absence of teeth leads to a reduction in these forces, resulting in bone resorption and changes in bone morphology [22]. Studies demonstrated that the rate and extent of resorption can vary significantly depending on several factors such as genetics, nutrition, age, gender, and time since tooth loss and oral hygiene which play roles in the degree of bone resorption observed. Also, suggesting that hormonal influences and metabolic rates could impact bone resorption [23]. Moreover, after the initial extraction of teeth, studies have shown the average first‐year bone loss is more than 1 mm in height and 50% in crestal bone width [24]. Then bone loss gets slower after the first year, but continuous throughout life [25].

Tan et al. [8] found a significant difference in CAn and sex difference, we found it in CAr which clearly affected by smaller bone ridge dimensions for female (Tables S3). From another side, in our study U type bone shape CAn mean was 61.2°, 61.1° in females and 63.7° in males. Other studies showed different values (Table S3), like a study done by Herranz‐Aparicio et al. it was 66.6° in males and 71.6° in females and the linear concavity depth 4.5 mm in males and 3.1 mm in females [13]. These differences could be attributed to the measurement’s calibration methods in different studies.

During the evaluation of morphological characteristics of mandibular lingual concavities, several distinct concavity criteria were identified:

1. Concavity position

Our findings revealed most of the lingual concavities were at MC level or above which make up higher risks for lingual plate perforation and require careful planning to avoid complications.

2. Concavity shape
  a. Steep and deep concavities: A smaller CAn indicates a steeper concavity in the bone structure. However, other parameters should be considered like bone ridge width and the CAr magnitude. The CAr magnitude perceives the depth and extent of the lingual concavity. A larger CAr can increase the complexity of implant placement, as it affects the angulation and positioning of the implant. Steep and deep concavities present higher risks for lingual plate perforation and require careful planning to avoid complications.
  b. Shallow and wide concavities: These generally provide more favorable conditions for implant placement but still require precise measurements to ensure optimal outcomes.

CBCT scanning was used in this study since it provides 3D information, including cross‐sectional morphology of the mandible, which cannot be obtained with periapical film or panoramic radiography, which only offers 2D view [9, 26]. In cadaver models the accuracy of CBCT and periapical films in prevention of implant‐related injury of the inferior alveolar nerve incidence was compared. When using periapical films for final implant drills insertion, damage to the mandibular inferior alveolar nerve was found in 31.8% of cases, whereas using CBCT revealed damage in 4.5% of cases [27]. Compared to traditional 2D imaging techniques, 3D imaging significantly reduces the risk of implant failure and postoperative complications. Furthermore, the risk of lingual plate perforation and inferior alveolar nerve injury were also assessed [3] using these models by providing a comprehensive view of the mandibular structure. Regarding these findings, CBCT was the technique of choice in this study to allow precise measurement of posterior mandible bone shape variations and lingual concavities.

The implications of our findings for clinical practice are significant. First, they emphasize the need for thorough preoperative planning using high‐resolution 3D imaging modalities such as CBCT. These technologies provide a detailed view of the mandibular anatomy, allowing for accurate assessment of lingual concavities. Our study, as well, supports the use of computer‐aided design and manufacturing (CAD/CAM) in creating surgical guides designed to the patient’s unique anatomy, thereby increasing the precision of implant placement. Further, we suggest that in cases of deep concavities, alternative surgical approaches or implant designs could be suggested. For example, shorter or angulated implants could be considered to avoid the risk of lingual plate perforation [15]. Additionally, bone grafting procedures may be needed to modify the bone morphology [20], creating a more favorable site for implant placement. While our study provides valuable insights, several limitations should be acknowledged. The demographic diversity may limit the generalizability of the findings. Future research should include more diverse cohorts to validate these results further. Another limitation is the static nature of our analysis. Dynamic factors such as opposing tooth and masticatory forces and their impact on bone morphology were not considered. Future studies could provide a more comprehensive understanding of how these factors could incorporate with the anatomical features of the mandible and future prosthetic replacement. Continued research and training in this area are essential to advance the field of dental implantology.

## 5. Conclusions

Comprehensive analysis of mandibular bone and lingual concavities is crucial for effective dental implant planning in the posterior mandible. U type bone ridge was the predominant bone shape in posterior mandible. 2nd molar sites showed more and relatively larger lingual concavities. Gender diversity created bone dimensions differences in which males showed bigger bone volume and larger lingual concavities. Tooth loss played significant role on bone ridge morphology over the time. The study highlights the importance of utilizing advanced imaging techniques to understand anatomical variations. By incorporating these findings into clinical practice, dental professionals can improve implant success rates and patient outcomes.

## Disclosure

All authors revised the manuscript for important intellectual content and approved the final version of the article.

## Conflicts of Interest

The authors declare no conflicts of interest.

## Author Contributions

Study conceptualization: Khulood Ali Al‐Taezi and Chunbo Tang. Data curation and analysis: Khulood Ali Al‐Taezi, Wei Chen, and Lin Liu. Manuscript drafting and editing: Zhankun Cai and Belal O M Muhaisen.

## Funding

This work was supported by the Jiangsu stomatological affiliated hospital of Nanjing Medical University.

## Supporting Information

Additional supporting information can be found online in the Supporting Information section.

## Supporting information


**Supporting Information** The supporting information accompany this manuscript and provide additional data supporting our findings: Figure S1: Alveolar ridge dimensions and mean differences between dentate and missing areas in 36, 37, 46, and 47 (*p* value < 0.05). Description: The figure illustrates the comparative alveolar ridge widths across these regions, highlighting statistically significant differences (*p* < 0.05) between dentate and missing sites. Table S1: Mean values of bone ridge width at different points of measurement in comparison to other studies. Description: The table presents mean ridge width values (1, 2, 3, 5 mm below crest and near the mandibular canal) for the first and second molar regions. It enables comparison with data from prior published studies to contextualize our findings. Table S2: Mean values of bone ridge height at different points of measurement in comparison to other studies. Description: The table details mean total ridge height and height measurements relative to the mandibular canal for molar regions, stratified by gender. It includes comparisons to previously reported values. Table S3: Mean values of lingual concavity parameters among different studies. Description: The table summarizes measured lingual concavity parameters for first and second molars, including gender differences and compares these values to data from the literature.

## Data Availability

The data that support the findings of this study are available from the corresponding author upon reasonable request.
