# Supplementary material for: Lingual Concavities in Posterior Mandible: Retrospective Morphometric Analysis in Relation to Gender and Tooth Loss
Source: Int J Dent. 2025 Dec 22;2025:5209739. doi: 10.1155/ijod/5209739 (PMC12752905; doi:10.1155/ijod/5209739)
Supplement: Supplementary file 1 — Supporting Information The supporting information accompany this manuscript and provide additional data supporting our findings: Figure S1: Alveolar ridge dimensions and mean differences between dentate and missing areas in 36, 37, 46, and 47 (p value < 0.05). Description: The figure illustrates the comparative alveolar ridge widths across these regions, highlighting statistically significant differences (p < 0.05) between dentate and missing sites. Table S1: Mean values of bone ridge width at different points of measurement in comparison to other studies. Description: The table presents mean ridge width values (1, 2, 3, 5 mm below crest and near the mandibular canal) for the first and second molar regions. It enables comparison with data from prior published studies to contextualize our findings. Table S2: Mean values of bone ridge height at different points of measurement in comparison to other studies. Description: The table details mean total ridge height and height measurements relative to the mandibular canal for molar regions, stratified by gender. It includes comparisons to previously reported values. Table S3: Mean values of lingual concavity parameters among different studies. Description: The table summarizes measured lingual concavity parameters for first and second molars, including gender differences and compares these values to data from the literature. [file IJOD-2025-5209739-s001.docx]

**Supplemantray materials**

**Figure 1.** Alveolar ridge dimensions and mean differences between dentate and missing areas in 36,37,46,and 47 (P value < 0.05).

**Table 1. Mean values of bone ridge width at different points of measurement in comparison to other studies**

| **Bone ridge width** | | | | | | | | | | | |  | |  |
| --- | --- | --- | --- | --- | --- | --- | --- | --- | --- | --- | --- | --- | --- | --- |
| **Region** |  | 1-2 mm | | 3 mm | | | 5 mm | | | Coronal 1/3 part | | Width 0-2mm superior to MC | | |
| **1^st^ molar** |  | F | M | F | M | | F | | M | F | M | F | M | |
| This study | Lft | 8.1 ±2.8 | 9.4 ±3.4* | 10.8 ±2.8 | 12.1 ±3.1* | | 12.6 ±2.7 | | 14.0 ±2.6* |  | | 14.2 ±2.0 | 14.8±2.6* | |
|  | Rt | 8.1 ±3.2 | 8.6 ±3.5* | 11.6 ±3.2 | 12.3 ±3.5* | | 13.8 ±2.8 | | 14.2 ±3.2 |  | | 14.9±2.0 | 15.8±2.5* | |
| Alqutaibi  et al^1^ | Lft | 11.5 ±1.4 | 11.3 ±1.1 |  | | |  | | | | | 13.2 ± 1.4 | 10.8 ± 2.3* | |
|  | Rt | 11.4 ±1.3 | 11.2 ±0.4 |  | | |  | | | | | 12.9 ± 2.2 | 11.4 ± 2.2* | |
| Saeed et al^2^ |  | 7.1 ±2.7 | 7.9 ±2.9* | 9.3 ±1.9 | 10.5 ±1.8* | | 10.9 ±1.5 | | 11.9 ±1.5* |  | |  |  | |
| Zhang et al^3^ |  |  |  |  | | |  | | | 10.2 ±2.3* | 11.7 ±2.7* | 10.4 ±1.9 | 11.5 ±2.1* | |
| Chan et al^4^ |  | 6.9 ±2 | 7.8 ±2.1* |  | | |  | | |  |  | 10.6±2.1 | 10.8 ± 2.0 | |
| Magat et al^5^ |  | 7.2 ±1.9 | 7.9 ±2.2 |  | | |  | | |  |  | 10.6 ±1.8 | 10.9 ±1.9 | |
| Herranz-Aparicio et al^6^ |  | 9.7 ±1.6 | 10.6 ±2.1* |  | | |  | | |  |  | 11.5 ±2.1 | 11.8 ±2.7* | |
| Bressan et al (16 mm posterior to mental foramen i.e. 1^st^ molar distal root)^7^ |  | 5.3 ±2.4 | 5.3 ±2.0 | 9.7 ±2.6 | | 9.2 ±2.2 | 11.4 ±2.0 | 11.3 ±2.2 | |  |  |  |  | |
| **2^nd^ molar** | |  |  |  | | |  | | |  | |  |  | |
| This study | Lft | 9.9 ±3.7 | 9.9 ±3.1 | 14.7 ±3.3 | 13.6 ±3.0 | | 17.3 ±1.7 | | 16.1 ±2.5 |  | | 15.8±2.3 | 16.0±2.6 | |
|  | Rt | 11.1 ±4.7 | 11.0 ±4.4 | 14.7 ±4.0 | 15.2 ±3.2 | | 16.8 ±2.4 | | 17.1 ±2.4 |  | | 16.8±2.5 | 16.5±2.8 | |
| Alqutaibi  et al | Lft | 15.1 ±2.8 | 12.7 ±1.6* |  | | |  | | |  | | 14.2 ± 4 | 11.9 ± 2.5* | |
|  | Rt | 14.6 ±2.5 | 12.7 ±1.6* |  | | |  | | |  | | 14.0 ± 2.4 | 12.5 ± 2.6* | |
| Saeed et al |  | 7.8 ±2.8 | 9.1 ±3.2* | 10.8 ±2.0 | 12.1 ±2.2* | | 12.9 ±2.0 | | 13.9 ±1.9* |  | |  |  | |
| Zhang et al |  |  |  |  | | |  | | | 10.7 ±2.4* | 12.7 ±2.1* | 11.1 ±2.3 | 11.9 ±2.4 | |
| **Molar region** |  |  |  |  | | |  | | |  |  |  |  | |
| Nickenig et al^8^ |  | 7.6 | |  | | |  | | |  |  | 11.0 | | |

*** Significant by gender**

**Table 2. Mean values of bone ridge height at different points of measurement in comparison to other studies**

| **Bone ridge height** | | | | | | | |
| --- | --- | --- | --- | --- | --- | --- | --- |
| **Region** |  | Total height | | | | Height 0-2 mm superior to MC | |
| **1^st^ molar** |  | F | | | M | F | M |
| This study | Lft | 28.5 ±3.0 | | | 31.1 ±3.3* | 16.5 ±2.3 | 17.1 ±2.7* |
|  | Rt | 28.3 ±3.1 | | | 31.1 ±3.3* | 16.6 ±2.7 | 18.2 ±3.0* |
| Alqutaibi et al | Lft |  | | |  | 12.2 ± 2.2 | 13.8 ± 3.1* |
|  | Rt |  | | |  | 12.8 ± 2.3 | 13.5 ± 2.4 |
| Saeed et al |  |  | | |  | 15.2 ±3.1 | 16.1 ±2.8* |
| Zhang et al |  | 26.3 ±2.8 | 28.8 ±3.2* | | | 15.5 ±2.2 | 17.6 ±3.3* |
| Chan et al |  |  | | |  | 12.2 ±2.4 | 12.8 ±3.1 |
| Magat et al |  |  | | |  | 13.3 ±2.7 | 14.8 ±3.0* |
| Herranz-Aparicio et al |  |  | | |  | 13.5 ±2.4 | 14.6 ±2.8* |
| Bressan et al |  |  | | |  | 8.9 ±3.0 | 11.5 ±3.0* |
| **2^nd^ molar** |  |  | | |  |  | |
| This study | Lft | 26.3 ±2.2 | | | 29.7 ±4.0 | 14.5 ±1.7 | 16.9 ±3.1* |
|  | Rt | 25.9 ±2.4 | | | 30.2 ±4.7 | 17.1 ±3.7 | 18.2 ±3.0* |
| Alqutaibi  et al | Lft |  | | |  | 10.5 ± 2.4 | 11.5 ± 2.9* |
|  | Rt |  | | |  | 10.7 ± 2.4 | 11.5 ± 2.6* |
| Saeed et al |  |  | | |  | 13.9 ±3.5 | 14.46 ±3.1 |
| Zhang et al |  | 23.3 ±2.4 | | 26.0 ±2.8* | | 14.1 ±1.9 | 15.6 ±2.7* |
| **Molar region** |  |  | |  | |  |  |
| Nickenig et al | 25.7 | | |  | | 13.1 | |

*** Significant by gender**

**Table 3. Mean values of lingual concavity parameters among different studies**

| **Lingual concavity** | | | | | | | | |
| --- | --- | --- | --- | --- | --- | --- | --- | --- |
| **Region** |  | CAn **(xº)** | | | LCD (mm) | | | |
| **1^st^ molar** |  | F | | M | F | M | | |
| This study | Lft | 66.6 ±11.5 | | |  |  | | |
|  | Rt | 70.8 ±11.3 | | |  |  | | |
| Alqutaibi et al | Lft | 52.7 ±12.8 | | | 2.1 ± 0.8 | | | |
|  | Rt | 52.0 ±16.3 | | | 1.8 ± 0.8**⁺** | | | |
| Chan et al |  | 56.8 ±12 | | 59.3 ±7.3 | 2.4 1.1 | | 2.4 1.1 | |
| Magat et al |  | 64.3 ±9.7 | | 62.5 ±7.1 | 2.6 ±0.9 | | 3.2 ±0.9* | |
| Herranz-Aparicio et al |  | 71.6 ±8.4 | | 66.6 ±8.9* | 3.1 ±1.7 | | 4.5 ±2.3* | |
| **2^nd^ molar** |  |  | |  |  | | | |
| This study | Lft | 58.5 ±12.0 | | |  |  | | |
|  | Rt | 64.6 ±8.7 | | |  |  | | |
| Alqutaibi et al | Lft | 44.1 ±8.8 | | | 2.0 ± 0.8 | | | |
|  | Rt | 43.5 ±8.9 | | | 2.2 ± 0.7**⁺** | | | |
| **Molars region** |  |  |  | |  | | |  |
| Nickenig et al |  | 53.9 | | | 3.7 | | | |

*** Significant by gender**

**⁺ Significant by tooth side**

**Supplementary Materials**

The supplementary materials accompany this manuscript and provide additional data supporting our findings:

**Supplementary Figure 1:**

- **Title:**Alveolar ridge dimensions and mean differences between dentate and missing areas in 36,37,46, and 47 (P value < 0.05).
- **Description:** This figure illustrates the comparative alveolar ridge widths across these regions, highlighting statistically significant differences (p < 0.05) between dentate and missing sites.

**Supplementary Table 1:**

- **Title:**Mean values of bone ridge width at different points of measurement in comparison to other studies
- **Description:** This table presents mean ridge width values (1–2 mm, 3 mm, 5 mm below crest and near the mandibular canal) for the first and second molar regions. It enables comparison with data from prior published studies to contextualize our findings.

**Supplementary Table 2:**

- **Title:** Mean values of bone ridge height at different points of measurement in comparison to other studies
- **Description:** This table details mean total ridge height and height measurements relative to the mandibular canal for molar regions, stratified by gender. It includes comparisons to previously reported values.

**Supplementary Table 3:**

- **Title:** Mean values of lingual concavity parameters among different studies.
- **Description:** This table summarizes measured lingual concavity parameters for first and second molars, including gender differences, and compares these values to data from the literature.

**References:**

1. Alqutaibi AY, Alghauli MA, Aboalrejal A, et al. Quantitative and qualitative 3D analysis of mandibular lingual concavities: Implications for dental implant planning in the posterior mandible. *Clin Exp Dent Res*. 2024;10(1). doi:10.1002/cre2.858

2. Saeed TA, Alansy AS, Abdu ZA, Almaqtari O, Yu Z. Dentulous versus edentulous mandibles: CBCT-based morphometric assessment of mandibular canal and alveolar bone. *J Clin Exp Dent*. 2022;14(12):986-993. doi:10.4317/jced.59033

3. Zhang W, Tullis J, Weltman R. Cone beam computerized tomography measurement of alveolar ridge at posterior mandible for implant graft estimation. *Journal of Oral Implantology*. 2015;41(6):e231-e237. doi:10.1563/aaid-joi-D-14-00146

4. Chan HL, Brooks SL, Fu JH, Yeh CY, Rudek I, Wang HL. Cross-sectional analysis of the mandibular lingual concavity using cone beam computed tomography. *Clin Oral Implants Res*. 2011;22(2):201-206. doi:10.1111/j.1600-0501.2010.02018.x

5. Magat G. Radiomorphometric analysis of edentulous posterior mandibular ridges in the first molar region: A cone-beam computed tomography study. *J Periodontal Implant Sci*. 2020;50(1):28-37. doi:10.5051/jpis.2020.50.1.28

6. Herranz-Aparicio J, Marques J, Almendros-Marqués N, Gay-Escoda C. Retrospective study of the bone morphology in the posterior mandibular region. Evaluation of the prevalence and the degree of lingual concavity and their possible complications. *Med Oral Patol Oral Cir Bucal*. 2016;21(6):e731-e736. doi:10.4317/medoral.21256

7. Bressan E, Ferrarese N, Pramstraller M, Lops Di, Farina R, Tomasi C. Ridge Dimensions of the Edentulous Mandible in Posterior Sextants: An Observational Study on Cone Beam Computed Tomography Radiographs. *Implant Dent*. 2017;26(1):66-72. doi:10.1097/ID.0000000000000489

8. Nickenig HJ, Wichmann M, Eitner S, Zöller JE, Kreppel M. Lingual concavities in the mandible: A morphological study using cross-sectional analysis determined by CBCT. *Journal of Cranio-Maxillofacial Surgery*. 2015;43(2):254-259. doi:10.1016/j.jcms.2014.11.018
